# Establishment of Rat Embryonic Stem Cells and Making of Chimera Rats

**DOI:** 10.1371/journal.pone.0002800

**Published:** 2008-07-30

**Authors:** Shinobu Ueda, Masaki Kawamata, Takumi Teratani, Taku Shimizu, Yoshitaka Tamai, Hiromasa Ogawa, Katsuyuki Hayashi, Hiroyuki Tsuda, Takahiro Ochiya

**Affiliations:** 1 Section for Studies on Metastasis, National Cancer Center Research Institute, Tokyo, Japan; 2 Comprehensive Research Organization, Waseda University, Tokyo, Japan; 3 BANYU, Tsukuba Research Institute, Tsukuba, Japan; 4 CLEA Japan, Inc., Tokyo, Japan; 5 DNA Chip Research, Inc., Yokohama, Japan; 6 Department of Molecular Toxicology, Nagoya City University Graduate School of Medical Sciences, Nagoya, Japan; Netherlands Cancer Institute, Netherlands

## Abstract

The rat is a reference animal model for physiological studies and for the analysis of multigenic human diseases such as hypertension, diabetes, neurological disorders, and cancer. The rats have long been used in extensive chemical carcinogenesis studies. Thus, the rat embryonic stem (rES) cell is an important resource for the study of disease models. Attempts to derive ES cells from various mammals, including the rat, have not succeeded. Here we have established two independent rES cells from Wister rat blastocysts that have undifferentiated characters such as Nanog and Oct3/4 genes expression and they have stage-specific embryonic antigen (SSEA) -1, -3, -4, and TRA-1-81 expression. The cells were successfully cultured in an undifferentiated state and can be possible over 18 passages with maintaining more than 40% of normal karyotype. Their pluripotent potential was confirmed by the differentiation into derivatives of the endoderm, mesoderm, and ectoderm. Most importantly, the rES cells are capable of producing chimera rats. Therefore, we established pluripotent rES cell lines that are widely used to produce genetically modified experimental rats for study of human diseases.

## Introduction

ES cells are derived from the inner cell mass (ICM) of blastocysts and are capable of unlimited, undifferentiated proliferation *in vitro*. Mouse ES cell (mES) lines were first established by culturing ICM [1], [2] in the presence of the feeder cell layer made of mouse embryonic fibroblast (MEF) and leukemia inhibitory factors (LIF) [3]. These cells have a stable developmental potential to form derivatives of all three embryonic germ layers even after prolonged culture [4], and have been used to study the mechanism of cell differentiation. Moreover, they are capable of generating germ-line chimeras following injection into the blastocyst [5]. Thus, the creation of targeted mutation in the mouse has been a valuable source of animal models of human disease.

The rat is a reference animal model for physiological studies and for the analysis of multigenic human diseases such as hypertension, diabetes, and neurological disorders [6] and in extensive chemical carcinogenesis studies, rats have been used for a long period. Thus, rES cells are important resources for the study of disease models.

Previously, attempts to establish a stable pluripotent ES cell line from rats have been reported [7], [8], [9], [10], [11]. However, the developmental potentials of these cell lines remain poorly characterized and they have not yet been shown to reconstitute the germ line.

Here we have established rES cells that have all undifferentiated characters, a stable long-term culture condition, maintain a normal karyotype, maintain a pluripotent potential to differentiate into derivatives of the endoderm, mesoderm, and ectoderm, and most importantly, that are capable of producing chimeras.

Thus, our rES cells will provide a useful resource for the development of large animal models for human disease, regenerative medicine, and pharmaceutical research.

## Results

### Establishment and Maintenance of Rat ES Cell Lines

**Figure 1:**
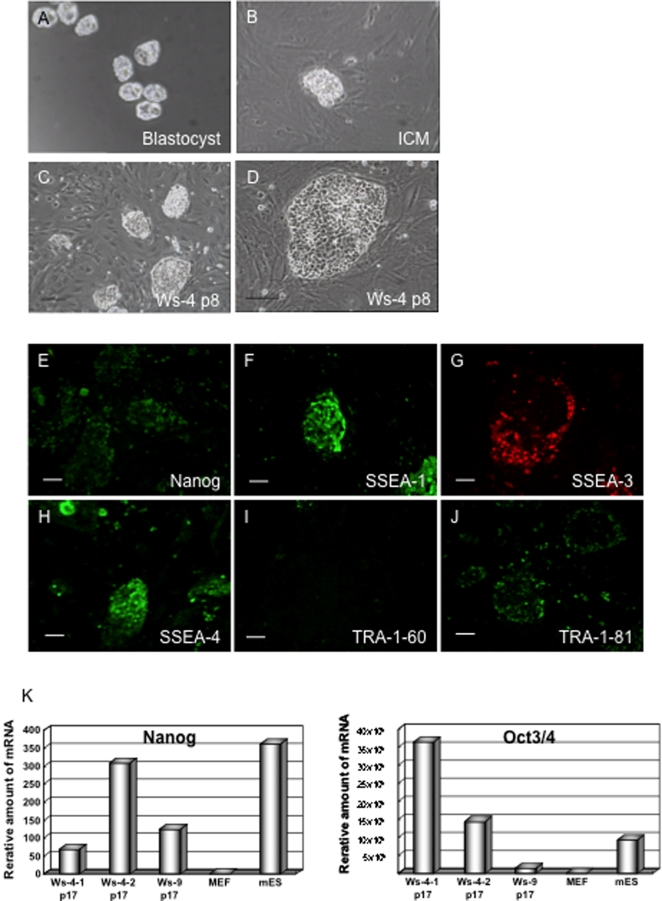
Generation of rat ES cells and expression of stem cell markers. Frozen Jcl:Wister blastocysts were treated in tyrode's Solution. (B) ICM derived cells attached to MEFs after 3 days of blastocyst culture and (C and D) Ws-4 colonies at passage 8. Scale bar, 200 µm. Expression of cell surface markers by Ws-4-2 cells at passage 13. (E) Nanog, (F) SSEA-1, (G) SSEA-3, (H) SSEA-4 and (J) TRA-1-81 were positive. (I) TRA-1-60 was negative. Scale bar, 100 µm. (K) Nanog and Oct-3/4 expression analysis of rES cells. Transcript levels were normalized to rat GAPDH expression, with expression levels MEFs set as 1.0. Real-time PCR analysis of Ws-4-1 p17, Ws-4-2 p17, Ws-9 p17, MEFs and mES (derived from 129sv). Nanog (Left) and Oct-3/4 (Right) were detected in all rES cell lines; however, each rES cell lines shows different expression levels.

Zona-free blastocysts or morulae embryos (Figure 1A) were cultured on a feeder layer for 2 or 3 days. As a result, total 42 ICM showed enough growth to be subcultured in rES culture medium (RESM) (Figure 1B). Rat LIF was not absolutely required during the process of ICM growth. These ICM were dissociated into clusters of cells. These clusters derived from some embryos were mixed and transferred onto a new feeder layer for propagation, because the cells needed to be high density to establish rES cell lines. Several days after subculturing, colonies with highly compacted cell clusters were picked up and transferred onto a new feeder layer. rES cell lines were stably maintained by the bulk passage method [12] using both collagenase treatment and disaggregation of colonies by pipetting using Pipetman P-1000. The cells were typically subcultured every 3–4 days at a splitting ratio of 1∶3. After these procedures two independent ES cell lines (Ws-4 and Ws-9) (Figures 1C and D, Figures S1A and B) were isolated, successfully propagated and used for detailed analyses. Ws-4 rES cells were maintained in the presence of rat LIF at 1000 U/ml up to passage 5 and then they were divided into three groups: Ws-4-1 was cultured in the presence of 250 U/ml rat LIF; Ws-4-2 was cultured in the presence of 500 U/ml rat LIF; Ws-4-3 was cultured in the presence of 1000 U/ml rat LIF. Ws-9 was maintained without rat LIF. These cells grew flat and possessed a similar morphology to the human and monkey ES cell lines (Figures 1C and D, Figures S1A and B). They did not form domed colonies, which are typical for mES cells. Ws-4-3 cells were not used further analysis since they rapidly grew and easily lost their stem cell-like phenotype.

### Stem Cell Markers and Karyotype analysis of rES cells

To characterize the properties of the isolated cell lines, we analysed alkaline phosphatase activity, SSEA-1, SSEA-3, SSEA-4, TRA-1-60, TRA-1-81 and Nanog by immunostaining. Mouse ICM, ES cells and EC cells express SSEA-1 but do not express SSEA-3 or SSEA-4. Ws-4-2 rES cells expressed SSEA-1, SSEA-3, SSEA-4, TRA-1-81 and Nanog at passage13 (Figures 1E–H and J). However, these cells were negative for alkaline phosphatase activity (data not shown) and TRA-1-60 (Figure 1I). Ws-9 rES cells expressed SSEA-1, SSEA-3, SSEA-4, TRA-1-81 and Nanog at passage 12 (Figures S1C–F and H). Ws-9 rES cells were also negative for alkaline phosphatase activity (data not shown) and TRA-1-60 (Figures S1G). Their summary was presented in Table S1.

Next, we examined expression of several molecular markers by real-time PCR analysis. Like Oct3/4, the expression of Nanog mRNA is restricted to pluripotent stem cells and absent in differentiated ones [13], [14]. The Ws-4-1, Ws-4-2 and Ws-9 rES cells expressed Nanog and Oct3/4 at passage 17 (Figure 1K).

Karyotype was assessed during the process of establishment and long-term passage of rES cells. Chromosome number of each cell line was counted as shown in Figure S2 and their summary was presented in Table S2. The Ws-4-1, Ws-4-2, Ws-4-3 and Ws-9 rES cells had more than 40% of normal karyotype at passage 11 to 18.

### Differentiation Potency

**Figure 2:**
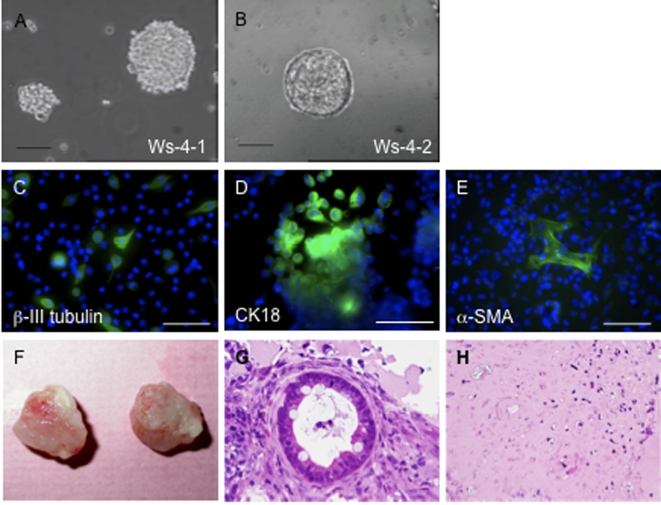
Pluripotency of rat ES cells. Embryoid bodies formation (A) Ws-4-1and (B) Ws-4-2. Scale bar, 200 µm. Immunostaining confirming *in vitro* differentiation into all three germ layers (Ws-4-1). Expression of (C) beta-III tubulin (ectoderm), (D) cytokeratin 18 (endoderm) and (E) alpha-SMA (mesoderm). Scale bar, 50 µm. (F) Ws-9 derived tumor after subcutaneous injection into SCID-mouse. Hematoxylin and eosin stained histological sections of rES (Ws-9) derived tumor: (G) intestinal epithelium-like structure (endoderm) and (H) cartilage-like structure (mesoderm).

EBs were produced by culturing rES cell aggregates in Petri dishes. Ws-4-1 and Ws-9 rES cells formed simple EBs within a few days (Figure 2A). Especially, when these cultures were maintained, simple EBs of Ws-4-2 developed cystic EBs (Figure 2B).

To examine rES cell differentiative capacity in greater detail, we determined whether rES cells could differentiate into a wide variety of cell types *in vitro*. Generation of derivatives of the three germ layers from Ws-4-1 was confirmed by the expression of markers including beta-III tubulin (neuroectoderm) (Figure 2C), nestin (Figure S3A), cytokeratin 18 (endoderm) (Figure 2D), alpha-smooth muscle actin (SMA) (mesoderm) (Figure 2E) and CD31 (Figure S3B). Ws-9 differentiated into ectoderm (beta-III tubulin, Figure S3C) and endoderm (CK18, Figure S3D). Mesoderm differentiation from Ws-9 was not tested. Next, we transplanted the rES cells of the Ws-4-1, Ws-4-2 and Ws-9 line respectively into the severe combined immunodeficient (SCID)-mice. Tumors formation was found after 5–16 weeks. Ws-4-1 formed tumors from all injections (subcutaneous injections: n = 3, and intratesticular injection: n = 1). Ws-4-2 formed a tumor from subcutaneous injection (n = 1). Ws-9 formed tumors from 3 subcutaneous injections (n = 5) and from 2 intraperitoneal injections (n = 6). The tumor in 6 weeks from Ws-9 was shown (Figure 2F as subcutaneous tumors). These tumors were consisted from two germ layers including an intestinal epithelium-like structure (endoderm, Figure 2G), and a cartilage-like structure (mesoderm, Figure 2H). An ectodermal origin structure was not found in these tumors.

### Making of Chimera Rats

**Figure 3:**
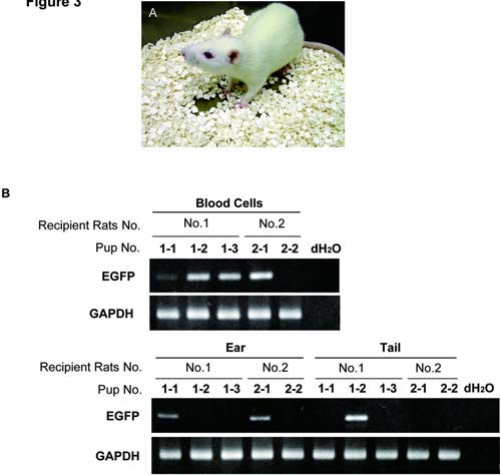
Contribution of rES cells to chimeras. (A) Live chimera rats derived from rES cells (Ws-4-2) were obtained. (B) Four chimeric rats were obtained from 2 pseudopregnant rats by genotyping of blood cells, ears and tails.

Next, we examined the ability of rES cells to produce chimeras. For this purpose, rES cell lines named Ws-4-1, Ws-4-2 and Ws-9 were used. We injected EGFP-positive rES cells (Figure S4B) into Jcl:Wister rat derived blastocysts, which we then transplanted into the uteri of pseudo-pregnant rats. We obtained total 11 pups born (7 alive and 4 dead) and 6 embryos (E18.5 or E15.5) from 351 injected and transferred blastocysts using three independent cell lines (Table 1). We examined the presence of chimera rats by PCR analyses to know whether rES cells contributed to various organs. This yielded 10 chimera rats (Figure 3 and Table 1). In 3 pups derived from Ws-4-1 rES cells, PCR analysis detected rES cell contribution in the liver, heart and skin (Figure S5A). In 4 live pups from Ws-4-2 rES cells, PCR analysis detected rES cell contribution in the blood cells, ears and tails (Figure 3B). We obtained 4 embryos at E18.5 from Ws-4-1 and 2 embryos at E15.5 from Ws-4-2, and PCR analysis detected rES cell contribution in 3 embryos (E18.5) from Ws-4-1 (Figure S5B). The chimera rats derived from Ws-4-2 rES cells were grown and showed no abnormality (Figure 3A). In rES cells of Ws-9 line, no chimera rat was obtained so far.

**Table 1:** Efficiency of making of chimera rats from rES cells.

	Injected Blastocysts	Pups*	No.of Screened Pups	Chimera
Ws-9	122	2	1	0
Ws-4-1	110	4	3	3
		4 (E18.5)	4	3
Ws-4-2	119	5	5	4
		2 (E15.5)	2	0

* Live pups, dead pups and developmentally abnormal embryos were included in “Pups.”

## Discussion

Here, we show for the first time that we have established the rES cells from a Wister rat blastocyst. Our rES cells have all undifferentiated characters as follows: 1) a stable long-term culture can be possible over 18 passages with maintaining more than 40% of normal karyotype; 2) express ES cell-specific genes such as Nanog and Oct3/4; 3) express SSEA-1, -3, -4, and TRA-1-81; 4) maintain the pluripotent potential to differentiate into derivatives of all three embryonic germ layers and are capable of producing chimera rats. Thus, these results define our rES cells as an embryonic stem cell line and will be routinely used to generate targeted mutations, conditional knockout and gene replacement, which are required to produce relevant large animal models for human physiology, disease, regenerative medicine, and pharmaceutical research.

We established and characterized two independent ES cell lines. Interestingly, the morphology of rES was similar to primate ES cells reported so far and the cells formed flatter colonies than the typical domed colonies of mES cells. Similar to primate ES cells, rES cells exhibited a very low plating efficiency when dissociated into single cells.

In the case of cynomolgus monkey ES cells, the differentiated cells were greatly reduced by replacement of FBS with KSR [15]. Furthermore, bFGF was reported to increase the cloning efficiency of human ES (hES) cells [16]. However, rES cells were not established and there were slower growth rates in the KSR medium than in the FBS medium with or without rat bFGF.

Maintenance of the undifferentiated state and pluripotency in mES cells requires the presence of MEF feeder layers or LIF. LIF is known to bind to its receptor, LIFR, which heterodimerizes with the signal-transducing receptor gp130. The pluripotency of mES depends on the intracellular signaling events that follow, including phosphorylation by the Janus family of tyrosine kinases (JAK), which leads to activation of the signal transducer protein STAT3 [17]. LIFR, IL6R and gp130 were expressed on hES cells, and, when stimulated with either LIF or IL-6, activated STAT3. However, augmented levels of gp130-stimulated STAT3 activation failed to maintain hES cells in the undifferentiated state, with the progressive loss of TRA-1-60, Nanog, and Oct4 expression [18]. LIFR was expressed on our rES cells (Figure S6). We cloned complete rat LIF cDNA and determined the nucleotide sequence [19] because of the failure of recombinant mouse LIF to keep the pluripotent phenotype of rES cells long term. Although rat LIF was not necessary to ICM growth and its expansion, rat LIF was important to keep the expression levels of Nanog as an equivalent to mES cells. Nanog was expressed at lower levels in line Ws-4-1 (250 U/ml rat LIF) and Ws-9 (0 U/ml rat LIF) than in mES cells. It is reported that a 50% increase or decrease in Oct3/4 proteins induces differentiation of ES cells [20]. Oct3/4 expression was dramatically increased in Ws-4-1 and decreased in Ws-9. These data suggest that rat LIF is useful for maintenance of the undifferentiated state and pluripotency in rES cells for a long-term period.

When mES cells are grown in a serum-free medium, BMP4 and LIF can indeed support ES cell line derivation, maintenance of pluripotency, chimera colonization and germline transmission properties [21]. We found the BMP4 receptor expressed on rES cells (Figure S6). We examined whether the addition of rat LIF and human BMP4 might contribute to self-renewal and an inhibition of differentiation of rES cells. Ws-9 was maintained without rat LIF. Ws-9 cells were plated on MEFs in the rES medium with rat LIF (500 U/ml), human BMP4 (10 ng/ml) or rat LIF plus human BMP4. After day 4 and day 8, rES cell status was confirmed by expression of mRNAs for ES cell specific transcription factors Nanog, Oct3/4 and Sox2. We found a significant induction of Oct3/4 expression in the presence of rat LIF alone as well as rat LIF plus BMP4 at day 8 in culture (Figure S7). In contrast, Sox2 expression was reduced by rat LIF alone as well as rat LIF plus human BMP4. Nanog expression was not increased by rat LIF, human BMP4 or rat LIF plus human BMP4. These data suggest that 500 U/ml of rat LIF and 10 ng/ml of human BMP4 are not sufficient to maintain our rES cells for an expression of Oct3/4, Nanog and Sox2.

Our rES cells expressed some stem cell markers. However, expression patterns of SSEA antigens in some species are different. The expression patterns of SSEA antigens in rat EC cells and ICM cells are not well documented. Ws-9 was negative for SSEA-1 and TRA-1-81 and stained weakly for Nanog at passage 30, but retained expression of SSEA-3 (data not shown). These results suggest that differentiated cells stained for SSEA-3.

Oct3/4, Sox2, and Nanog have been shown to function as core transcription factors in maintaining pluripotency [22], [23]. rES cells expressed Oct3/4 and Nanog. Curiously, alkaline phosphatase activity, which generally marks pluripotent cells was present in hES cells and mES cells, but disappeared abruptly upon culture and was undetectable in rES cells. Alkaline phosphatase activity persists in primordial germ cells [24], [25] in the gastrulating embryo [26] and in their *in vitro* derivatives, the embryonic germ cells. Genes associated with the germ line, including *Stella*, *Piwil2*, *Stra8* and *Dazl* were expressed by mES cells and significantly decreased or were not detected in hES cells. *Stella* and *Piwil2* were not increased in rES cells by microarray analyses (Figure S8B). However, the rES cells expressed *Vasa* (Figure S6), which is present in fetal and adult gonadal germ cells in both males and females and is most abundant in spermatocytes and mature oocytes [27], [28]. These data suggest that rES cells might have a differentiating ability to germ cell lineage.

Our rES cell lines had a normal number of chromosomes at early passages, but lead to the rapid accumulation of chromosomal abnormalities in late passages. High rates of aneuploidy are known to be observed in hES cells during passage in culture. In particular, trypsin-based solutions tend to cause over-dissociation of ES cells into single cells, and this may promote karyotypic abnormalities [29]. Previously, we succeeded in establishing rES cell lines from Wistar Hannover GALAS rat. The resulting Wistar Hannover GALAS rES cells had undifferentiated characters; however, these cells developed karyotypic abnormalities during passage in culture using trypsin (data not shown). These abnormalities are associated with a decreased capacity of the cells for germ line colonization in chimeras obtained after blastocyst injection [30], [31]. However, it is reported that karyotype and *in vitro* differentiating ability of mES cells influence their germ-line contribution in chimeric mice. That both exhibit approximately 40% of normal karyotype and show cystic EBs formation within 7 days after suspension culture is a possible indication of their germ-line transmitting ability in chimeric mice [32]. In our rES cells, Ws-4-1, Ws-4-2 at 11 to 13 passages and Ws-9 cell lines at 18 passages retained more than 40% of normal karyotype and all they formed EBs within a few days, indicating that they have a potency to contribute germ-line transmission. In fact, our rES cells differentiated into three germ layers *in vitro*: neurons, epithelial cells and smooth muscle cells. Examination of tissue differentiation in teratomas produced from rES cells revealed more wide differentiation potency.

Our rES cells can contribute to chimera formation when injected into blastocysts. For further analysis of germline transmission, 4 live chimeras derived from Ws-4-2 were mated with normal males or females. Because rES cells do not survive well as single cells, we examined their potential to contribute to embryogenesis through diploid (4∼16-cell, n = 54) aggregation and tetraploid (2∼8-cell, n = 38) aggregation using rES cell line Ws-9. When rES cells were aggregated with diploid or tetraploid and transferred to oviducts of recipient females, we obtained no pups (data not shown). To test a developmental potency, 89 zona-free 2-cell without rES cells were cultured for 3 days and transferred to oviducts of recipient females. But no pups were obtained. These results suggest that rat zona-free embryos cultured *in vitro* are able to develop to blastocyst stage; however, postimplantation development is incomplete. Aggregation methods may not be suitable for making chimera rats.

Recently, pluripotent stem cells were derived from the late epiblast layer of post-implantation (EpiSCs) mouse and rat embryos [33], [34]. They seem to correspond very closely to human rather than mES cells. The only exception is that EpiSCs lack alkaline phosphatase activity. They are LIF and BMP4-independent and require instead activin/nodal and FGF for efficient self-renewal. EpiSCs can contribute poorly to chimeras after blastocyst injection, but germline transmission was not observed. EpiSCs provide a valuable experimental system for determining whether distinctions between mouse and human ES cells reflect species differences or diverse temporal origins. The rES cells, which derived from ICM, have a morphology similar to hES cells. The rES cells are likely to improve our understanding of the differences between mouse and rat. To further characterize our rES cells, the pattern of gene expression in two dependent lines of rES cells and mES cells was assessed by whole genome expression arrays. As a result, whole genome cluster analysis was performed by sorting of 3943 altered genes. The expression level of each gene in the MMC treated MEF (for mES cells) or MMC treated rat embryonic fibroblast (REF: for rES cells) sample was set to 1.0. Cluster analysis showed that independently derived rES lines were similar (correlation coefficients: 0.95795) and distinct from mES cells (correlation coefficients: 0.12554)(Figure S8A). To validate the results of the microarray analysis, we focused on several genes associated with ICM, mES, EpiSC, hES [34]. Our data show that the expression level of up-regulated genes in mES cells such as *cdh1/E-cadherin*, *Otx2*, *Cldn6*, *Nodal*, *Sox17*, *Foxa2, Acvr2b, Pitx2* and *Dkk1* were also up-regulated in our rES cells. On the other hand, our data show that the genes significantly increased in mES cells such as *Dppa3/Stella, Fbxo15, Lefty2, Nr01/Dax1* and *Sox2* were slightly or not increased in rES cells (Figure S8B and Table S3). Thus, microarray analysis shows that the gene expression profile of our rES cells partially resembles mES cells. Furthermore, using a database (GEO accession number GSE7902), the expression pattern of rES cells was compared with that of mES cells and EpiSCs [34]. The cluster analyses of rES cells, mES cells and EpiSCs were performed by sorting 2866 altered genes common to rat and mouse. Cluster analysis by sorting 2866 altered genes showed that independently derived rES lines were similar (correlation coefficients: 0.80782). Cluster analysis showed that EpiSCs were not similar to mES cells (correlation coefficients: 0.42110) and distinct from rES cells (correlation coefficients: 0.22710). The cluster analysis of mES cells using a database showed that mES cells were not similar to rES cells (correlation coefficients: 0.36524) (data not shown).

In this report, we have established rES cell lines that maintain a pluripotent potential and a normal number of chromosomes. And we have obtained chimera rats derived from rES cells by genotyping. The ability of these cells to give rise to germline transmission is currently under investigation. Our results pave the way for more extensive genetic modifications such as conditional knock-out and gene replacement, which are required to produce relevant models of human diseases.

## Materials and Methods

### Rat ES medium

Rat ES culture medium (RESM) consisted of 1∶1 mixture of Dulbecco's modified Eagle's medium (DMEM) and Ham's nutrient mixture F-12 (DMEM/F-12, Gibco) supplemented with 3% fetal bovine serum (Gibco), 0.1 mM 2-mercaptoethanol (Sigma), 1% nonessential amino acid stock (Gibco), 2 mM L-glutamine (Gibco), 1 mM sodium pyruvate (Gibco), 1× antibiotic-antimycotic (Gibco), 1× nucleoside stock (Sigma) (100×: Adenosine (0.8 mg/ml), Guanosine (0.85 mg/ml), Cytidine (0.73 mg/ml) Uridine (0.73 mg/ml) and Thymidine (0.24 mg/ml)).

### Establishment and Maintenance of Rat ES Cell Lines

Frozen Jcl:Wister embryos were prepared by CLEA Japan, Inc. Blastocysts (n = 37) and morulae (n = 14) were treated in tyrode's Solution (Ark Resource Co. Ltd.) and washed with Quinn's Advantage Blastocyst Medium (SAGE In-Vitro Fertilization, Inc. A Cooper Surgical Company) with 3% KSR (Gibco) added. Seven to ten zona-free embryos per 60 mm dish were placed on mouse embryonic fibroblasts inactivated with mitomycin C (MEFs, Millipore) in RESM. After 2 or 3 days, ICM-derived cells were dissociated into clumps by exposure to 0.025% trypsin (cell line Ws-4) or 0.05% collagenase type IV (Gibco) in DMEM/F-12 and mechanical dissociation (cell line Ws-9) with a Stem Cell Cutting Tool (SWEMED Lab International AB; Billdal, Sweden), before transfer onto a new feeder layer. Colonies with stem cell-like morphology were picked up, dissociated with 0.05% collagenase type IV and dissociated mechanically, and transferred to a feeder layer for expansion. Once established, rES cells were routinely passaged every 3–4 days up to 5 passages in RESM in the presence of rat LIF (Millipore/Chemicon International) at 1000 U/ml, and then they were cultured in RESM supplemented with 250 U/ml, 500 U/ml or 1000 U/ml rat LIF (Ws-4 rES cell line) or without rat LIF (Ws-9 rES cell line). Counting of the chromosome number was carried out at passage 10 to 18.

### Histochemical Analysis of the Stem Cell Markers

Alkaline phosphatase activity was detected with Vector Blue substrate (Vector Labs). SSEA-1, SSEA-3, SSEA-4, TRA-1-60, TRA-1-81 (Millipore) and Nanog (ReproCELL, Japan) were detected by immunocytochemistry. Cells were fixed in 4% paraformaldehyde, then incubated for 1 hour in blocking solution (image iT, Invitrogen). Primary antibodies applied for overnight at 4°C. Secondary antibodies conjugated to Alexa fluorophores (Molecular Probe) at 1∶1000 dilution were incubated with cells for 1.5 hours at room temperature.

### Real-time-PCR

Total RNA was isolated using the ISOGEN (Nippongene, Tokyo, Japan) and samples were treated with DNase I (Takara). The cDNA was made from 2 µg of total RNA using Super Script III RT (Invitrogen) and oligo-dT primers (Invitrogen).

cDNAs were used for PCR using Platinum SYBR Green qPCR SuperMix UDG (Invitrogen). Optimization of the qRT-PCR reaction was performed according to the manufacture's instructions (PE Applied Biosystems, Tokyo, Japan). All quantitations were normalized to an endogeneous control GAPDH. Relative gene induction values were calculated according to the manufacture's instructions. The following primers were used: *Nanog*, forward, 5′-GCCCTGATTCTTCTAGCAAT-3′ and reverse, 5′-AGAACACAGTCCGCATCTT-3′; *Oct3/4*, forward, 5′-CATCTGCCGCTTCGAG-3′ and reverse, 5′-CTCAATGCTAGTCCGCTTTC-3′; rat *Gapdh*, forward, 5′-TTCAACGGCACAGTCAAGG-3′ and reverse, 5′-CATGGACTGTGGTCATGAG-3′.

### Analysis of Differentiation Potency

The developmental potential of rES cells was assessed *in vitro* by forming EBs. rES cell colonies were harvested with collagenase IV, gently triturated, and cultured for 5–10 days in suspension culture dishes (Zarstat) containing RESM without rat LIF.

For differentiation of rES cells into neural lineages, the EBs were maintained for 2–3 days in RESM without rat LIF and for 3–4 days in RESM with 10^−6^ M retinoic acid (Sigma). EBs were treated with 0.25% trypsin-EDTA. Dispersed cells were collected by centrifugation and replated on poly-L-ornithin (Sigma)-coated 4 well Lab Tech II chamber slides (BD) in B27 medium (DMEM/F12 [1∶1] supplemented with B27 supplement (Invitrogen), 5 mM HEPES buffer (Sigma), 2 µg/ml heparin (Sigma), antibiotic-antimycotic (Invitrogen) and 1% FBS.

For differentiation of rES cells into endoderm and mesoderm, rES cells were cultured in the absence of MEFs on a gelatin coated plate in RESM without rat LIF.

Cells were fixed in 4% paraformaldehyde in phosphate buffered saline (PBS) for 20 min at room temperature. Fixed cells were blocked for 1 hr at room temperature with PBS/0.1% BSA/10% normal goat serum/0.3% Triton X-100 for beta-III tubulin and alpha-SMA or PBS/0.1% BSA/10% normal goat serum for cytokeratin 18 (CK18) then incubated overnight with anti-beta-III tubulin (COVANCE) at 1∶2000, anti-alpha-SMA (SIGMA) at 1∶1000 or anti-CK18 antibody (Millipore) at 1∶50. Secondary antibodies conjugated to Alexa fluorophores (Molecular Probe) at 1∶1000 dilution were incubated with cells for 1.5 hours at room temperature. Stained cells were analysed using HS All-in-one Flourescence Microscope (KEYENCE Corporation, Japan) and ECLIPSE TE300 (Nikon Corporation, Japan).

The rES cells were also tested for their ability to form teratomas. Approximately 10^7^ rES cells were injected subcutaneously or 10^6^–8×10^6^ rES cells were injected intratesticulally or intraperitoneally into the C.B17/Icr-scid (scid/scid) mice (CLEA Japan, Inc.). Teratomas were excised when tumor formation become outwardly apparent. They were fixed in phosphate-buffered 10% formalin or acetone. And they were processed for paraffin embedding for histological observation.

Animal experiments in the present study were performed in compliance with the guidelines of the Institute for Laboratory Animal Research, National Cancer Center Research Institute.

### Lentivirus Transduction to ES Cells

Lentivirus-green fluorescent protein (LV-GFP), which carries the expression cassette for eGFP, was constructed with pLenti6 (Invitrogen). rES cells were infected with LV-GFP at an moi of 250 or 500 for overnight in a volume of 500 µl. EGFP positive colonies were selected and cultured on a new feeder layer.

### Blastocyst Injection

The blastocysts were recovered from the uterus of natural mated Jcl:Wister females at 4.5 days post coitum (d.p.c.). The blastocysts were placed in a drop of HEPES (Nakamedical) under mineral oil. rES cells were injected into the blastocyst cavity using a microinjection pipette. After injection, blastocysts were returned to mW medium (Mitsubishi Kagaku Iatron, Inc.) and placed at 37°C until transferred to recipient females. From 14 to 21 injected blastocysts were transferred to each uterine horn of 4.5 d.p.c. of pseudo-pregnant Jcl:Wister rats. In total, Ws-4-1, Ws-4-2, and Ws-9 rES cells were injected into 351 blastocysts and they were subjected to uterine transfer.

### Genotyping

Offspring were screened for the presence of the EGFP gene by PCR. Blood cells, ear and tail of pups and each tissue of neonates killed at 15.5 or 18.5 d.p.c. were lysed in 50 mM Tris-HCl, pH 8.2, containing 25 mM EDTA, 0.5% SDS, 10 mM NaCl and 1 mg/ml proteinase K. PCR was performed with the following conditions: EGFP, 95°C 30 s, 58°C 30 s, 72°C 20 s (40 cycles) and GAPDH, 95°C 30 s, 60°C 30 s, 72°C 30 s (25 cycles). Primers were EGFP, forward, 5′-CTGACCCTGAAGTTCATCTG-3′ and reverse, 5′-TCCTTGAAGTCGATGCCCTT-3′; rat *Gapdh*, forward, 5′-TTCAACGGCACAGTCAAGG-3′ and reverse, 5′-CATGGACTGTGGTCATGAG-3′.

## Supporting Information

Table S1 (0.04 MB DOC)

Table S2 (0.03 MB DOC)

Table S3 (0.07 MB DOC)

Figure S1 Generation of rat ES cells and expression of stem cell markers. (A and B) Ws-9 colonies at passage 11. Scale bar, 200 µm Expression of cell surface markers by Ws-9 cells at passage 12. (C) Nanog, (D) SSEA-1, (E) SSEA-3, (F) SSEA-4 and (H) TRA-1-81 were positive. (G) TRA-1-60 was negative. Scale Bar, 50 µm
(1.56 MB TIF)

Figure S2 The Ws-4-2 rES cell lines had a normal number of chromosomes.
(1.56 MB TIF)

Figure S3 The rES cells could differentiate into a wide variety of cell types in vitro. Immunostaining confirming in vitro differentiation. Expression of (A) nestin (ectoderm) and (B) CD31 (mesoderm) were observed in differentiated Ws-4-1. Expression of (C) β-III tubulin (ectoderm) and (D) CK18 (endoderm) were observed in differentiated Ws-9.
(1.56 MB TIF)

Figure S4 rES cells transducted by LV-GFP. (A and B) EGFP positive colonies (line Ws-9) were picked up and injected into the blastosysts. (C and D) Cystic EBs were produced by EGFP positive Ws-9 cells. Scale Bar: 100 µm. (E and F) EGFP positive rES (line Ws-9) produced tumors after intraperitoneal injection.
(1.56 MB TIF)

Figure S5 Contribution of rES cells (Ws-4-1 and Ws-9) by genotyping. (A) Three chimeric rats were obtained from Ws-4-1 and no chimeric rat was obtained from Ws-9. (B) Three chimeric embryos (E18.5) were obtained from Ws-4-1. pCAG-EGFP is a vector with EGFP gene, positive control.
(1.56 MB TIF)

Figure S6 RT-PCR analysis of rat Lifr and Vasa gene expression in rES cells. Total RNA was isolated using the ISOGEN (Nippongene, Tokyo, Japan) and samples were treated with DNase I (Takara). cDNA was made from 2 µg of total RNA using Super Script III RT (Invitrogen) and oligo-dT primers (Invitrogen). cDNA was amplified with TaKaRa Ex Taq Hot Start Version (Takara, Japan) using gene specific primers. The following primers were used: rat Lifr, forward, 5′-CTGTCATTGTTGGCGTGGTA-3′ and reverse, 5′-GATTCCAGGACTTCGACGTG-3′, 30 cycles; rat Vasa, forward, 5′-TTGGGCACTCAATTCGAC-3′ and reverse, 5′-AACTTCTTCATTTCGGGTCC-3′, 32 cycles; rat Bmpr1a, forward, 5′-CCATTTCCAGCCCTACATCA-3′ and reverse, 5′-TTCCAGCGGTTAGAGACGAT-3′, 35 cycles.
(1.56 MB TIF)

Figure S7 Oct3/4 and Sox2 expression analysis of rES cells in response to LIF, BMP4, or LIF plus BMP4. Ws-9 was maintained without rat LIF. The 5×10^4^/well of Ws-9 cells were plated on MEFs of gelatin coated 6 well plates in the RESM. Culture condition was divided into four groups 1) rat LIF (-) and human BMP4 (-), 2) rat LIF (-) and human BMP4 (10 ng/ml), 3) rat LIF (500 U/ml) and human BMP4 (-), 4) rat LIF (500 U/ml) and human BMP4 (10 ng/ml). After day 4, rES cells were passaged 5×10^4^/well. Real-time PCR analysis of Ws-9 p14 (day 4), p15 (day 8) and MEFs. cDNAs were used for PCR using Platinum SYBR Green qPCR SuperMix UDG (Invitrogen). Optimization of the qRT-PCR reaction was performed according to the manufacture's instructions (PE Applied Biosystems, Tokyo, Japan). The following primers were used: Oct3/4, forward, 5′-CATCTGCCGCTTCGAG-3′ and reverse, 5′-CTCAATGCTAGTCCGCTTTC-3′; Sox2, forward, 5′-CCCACCTACAGCATGTCCTA-3′ and reverse, 5′-TGGAGTGGGAGGAAGAGGTA-3′; rat Gapdh, forward, 5′-TTCAACGGCACAGTCAAGG-3′ and reverse, 5′-CATGGACTGTGGTCATGAG-3′. Transcript levels were normalized to rat GAPDH expression, and expression levels of rES cell cultured without rat LIF and human BMP4 at day 4 set to 1.0. The results present the mean of two real-time PCR analysis. Oct3/4 (Red) expression was increased in rat LIF and rat LIF + human BMP4, however, Sox2 (Blue) expression was not increased.
(1.56 MB TIF)

Figure S8 Microarray analysis and hierarchical clustering analysis. A one-color microarray-based gene expression analysis system (Agilent Technologies, Santa Clara, CA) containing 41,000 genes was used, following the manufacturer's instructions. Total RNA was extracted from MMC treated MEF, mES cells (derived from 129sv, p6), MMC treated rat embryonic fibroblast (REF, 3Y1-B,p5) [19], Ws-4-2 (p17), Ws-4-2 (p14) and Ws-9 (p17). The process of hybridization and washing was performed using a Gene Expression Wash Pack (Agilent Technologies) and acetonitrile (Sigma, Tokyo, Japan). A DNA microarray scanner (Agilent Technologies) was used for array scanning. Data normalization and cluster analysis were performed using GeneSpring GX software (Agilent Technologies). The expression level of each gene in the MMC treated MEF (for mES cells) or MMC treated REF (for rES cells) was used as a reference. Microarray data of mouse and rat was integrated via GeneSymbol, and omitted missing values. This resulted in a data matrix of 3943 genes. A hierarchical cluster was produced using an Euclidean distance calculation based on the Ward method calculation. (A) Cluster analysis was performed by sorting 3943 altered genes. Red indicates increased expression compared to levels of MMC treated MEF or MMC treated REF, whereas green means decreased expression. Correlation coefficients are indicated. This analysis of microarray data shows that there are distinct gene clusters between rES cells and mES cells. (B) The expression of genes to maintain the pluripotent state of mES cells or EpiSCs or hES cells. Gene names shown in red were detected in hES cell cultures and EpiSC using Illumina and Agilent whole-genome microarrays [34]. This microarray analysis shows that the gene expression profile of our rES cells partially resembles mES cells.
(1.56 MB TIF)
