# Supplementary material for: Establishment of Rat Embryonic Stem Cells and Making of Chimera Rats
Source: PLoS One. 2008 Jul 30;3(7):e2800. doi: 10.1371/journal.pone.0002800 (PMC2483735; doi:10.1371/journal.pone.0002800)
Supplement: Table S1 — (0.04 MB DOC) [file pone.0002800.s001.doc]

**Table S1. Stem cell markers**

|  | **Ws-4 p13** | **Ws-9 p12** |
| --- | --- | --- |
| **ALP**  **Nanog**  **SSEA-1**  **SSEA-3**  **SSEA-4**  **TRA-1-60**  **TRA-1-81** | **-**  **+**  **+**  **+**  **+**  **-**  **+** | **-**  **+**  **+**  **+**  **+**  **-**  **+** |
