# Supplementary material for: Establishment of Rat Embryonic Stem Cells and Making of Chimera Rats
Source: PLoS One. 2008 Jul 30;3(7):e2800. doi: 10.1371/journal.pone.0002800 (PMC2483735; doi:10.1371/journal.pone.0002800)
Supplement: Table S2 — (0.03 MB DOC) [file pone.0002800.s002.doc]

**Table S2. Chromosome number of each cell line**

| **Cell line Passage Normal/counted (%)** | | |
| --- | --- | --- |
| **Ws-4-1**  **Ws-4-1-GFP** | **p11**  **p17** | **23/50 (46)**  **11/30 (36.7)** |
| **Ws-4-2**  **Ws-4-2-GFP-1**  **Ws-4-2-GFP-2**  **Ws-4-2-GFP-3**  **Ws-4-2-GFP-4**  **Ws-4-2-GFP-5** | **p11**  **p13**  **p16**  **p16**  **p16**  **p16**  **p16** | **25/52 (48)**  **22/50 (44)**  **5/56 (8.9)**  **23/64 (35.9)**  **11/55 (20)**  **17/62 (27.4)**  **13/57 (22.8)** |
| **Ws-4-3** | **p13** | **24/50 (48)** |
| **Ws-9-GFP-1** | **p14** | **5/54 (9.3)** |
| **Ws-9-GFP-2**  **Ws-9-GFP-2-6** | **p14**  **p18** | **4/40 (10)**  **20/48 (41.7)** |
| **Ws-9-GFP-3** | **p14** | **8/52 (15.4)** |

Chromosome number of each cell line was counted at various passage numbers.

Diploid (42) chromosomes were counted as normal.
