# Supplementary material for: Establishment of Rat Embryonic Stem Cells and Making of Chimera Rats
Source: PLoS One. 2008 Jul 30;3(7):e2800. doi: 10.1371/journal.pone.0002800 (PMC2483735; doi:10.1371/journal.pone.0002800)
Supplement: Table S3 — (0.07 MB DOC) [file pone.0002800.s003.doc]

**Table S3. The relative gene expression of rES cells and mES cells**

| **Gene Symbos** | **MEF** | **mES**  **p6** | **REF** | **Ws-4-2 p17** | **Ws-4-2 p14** | **Ws-9** |
| --- | --- | --- | --- | --- | --- | --- |
| **Acvr2b** | 0.9999999 | 30.44 | 1 | 11.4 | 12.07 | 8.99 |
| **Cdh1/E-cadherin** | 0.9999999 | 2910.01 | 1 | 5147.36 | 3207.0 | 2895.70 |
| **Cldn6** | 1 | 282.995 | 0.9999999 | 72.87 | 56.80 | 93.40 |
| **Dkk1** | 1 | 6.035 | 1 | 120.2 | 25.85 | 38.68 |
| **Dppa3/Stella** | 1 | 547.66 | 1 | 0.83 | 0.74 | 1.27 |
| **Fbxo15** | 1 | 547.445 | 1 | 2.77 | 3.59 | 5.53 |
| **Foxa2** | 1 | 30.92 | 1 | 37.90 | 40.07 | 52.60 |
| **Gal** | 1 | 1.22 | 1 | 2.67 | 0.70 | 0.52 |
| **Gata6** | 1 | 1.93 | 1 | 19.62 | 21.07 | 19.28 |
| **Gbx2** | 1 | 2.28 | 1 | 2.81 | 1.84 | 1.93 |
| **Klf4** | 1 | 0.57 | 1 | 7.29 | 7.58 | 7.15 |
| **Lefty2** | 1 | 1118.75 | 1 | 15.37 | 5.41 | 2.32 |
| **Myc** | 1 | 0.81 | 1 | 1.05 | 0.26 | 0.42 |
| **Nodal** | 1 | 123.395 | 1 | 7.04 | 3.72 | 16.45 |
| **Nr0b1/Dax1** | 1 | 1113.975 | 1 | 0.59 | 0.63 | 0.52 |
| **Otx2** | 1 | 389.505 | 1 | 1748.22 | 392.39 | 148.62 |
| **Pecam1** | 1 | 0.795 | 1 | 1.22 | 2.27 | 3.72 |
| **pitx2** | 1 | 8.65 | 1 | 303.64 | 175.67 | 733.68 |
| **Piwil2** | 1 | 12.615 | 1 | 1.04 | 0.96 | 0.79 |
| **Sox17** | 1 | 61.31 | 1 | 101.67 | 59.70 | 71.07 |
| **Sox2** | 1 | 1697.51 | 1 | 1.09 | 0.62 | 2.35 |
| **Tbx3** | 1 | 3.785 | 1 | 5.15 | 3.37 | 3.18 |

The expression level of each gene in the MMC treated MEF (for mES cells) or MMC treated REF (for rES cells) sample was set to 1.0.
