# Supplementary figures and images for: Establishment of Rat Embryonic Stem Cells and Making of Chimera Rats
Source: PLoS One. 2008 Jul 30;3(7):e2800. doi: 10.1371/journal.pone.0002800 (PMC2483735; doi:10.1371/journal.pone.0002800)

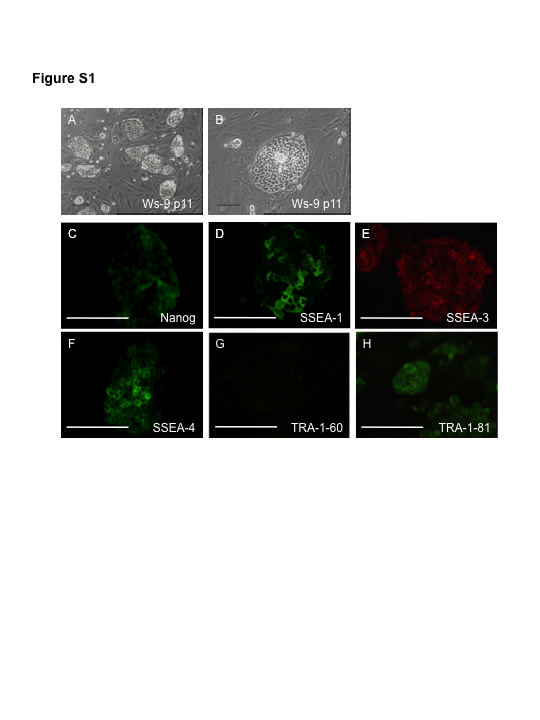

Supplement: Figure S1 — Generation of rat ES cells and expression of stem cell markers. (A and B) Ws-9 colonies at passage 11. Scale bar, 200 µm Expression of cell surface markers by Ws-9 cells at passage 12. (C) Nanog, (D) SSEA-1, (E) SSEA-3, (F) SSEA-4 and (H) TRA-1-81 were positive. (G) TRA-1-60 was negative. Scale Bar, 50 µm (1.56 MB TIF) [file pone.0002800.s004.tif]

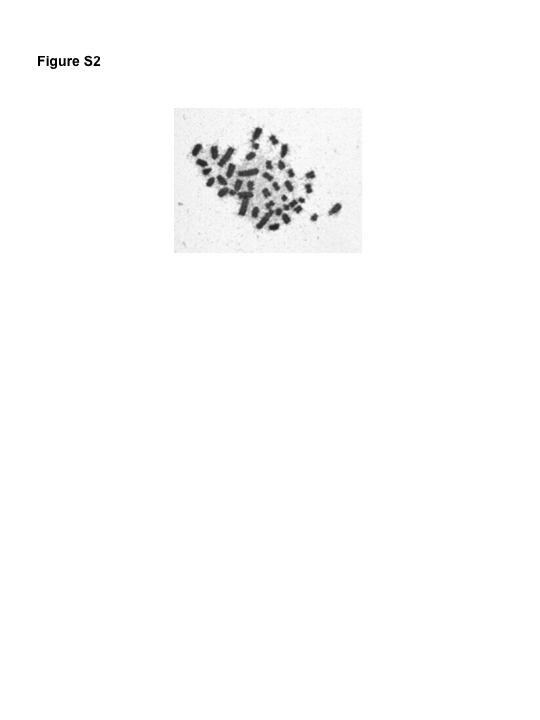

Supplement: Figure S2 — The Ws-4-2 rES cell lines had a normal number of chromosomes. (1.56 MB TIF) [file pone.0002800.s005.tif]

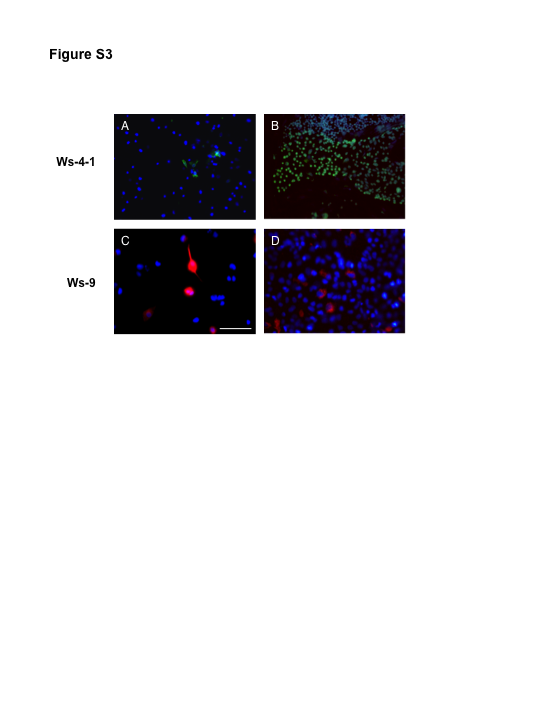

Supplement: Figure S3 — The rES cells could differentiate into a wide variety of cell types in vitro. Immunostaining confirming in vitro differentiation. Expression of (A) nestin (ectoderm) and (B) CD31 (mesoderm) were observed in differentiated Ws-4-1. Expression of (C) β-III tubulin (ectoderm) and (D) CK18 (endoderm) were observed in differentiated Ws-9. (1.56 MB TIF) [file pone.0002800.s006.tif]

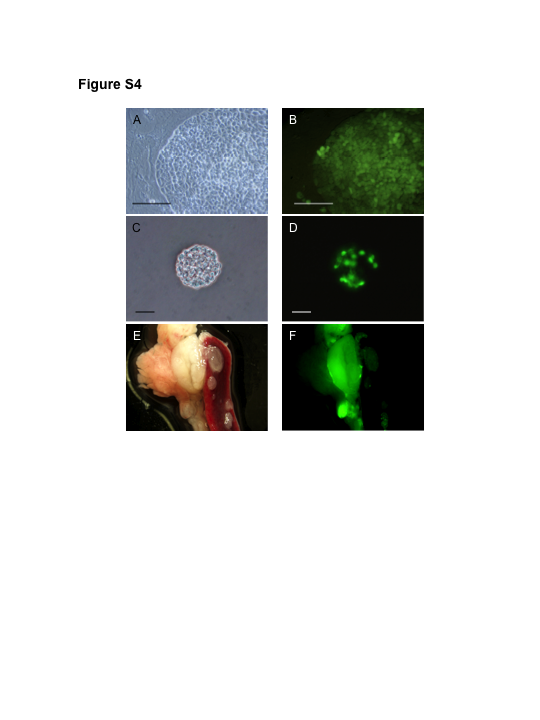

Supplement: Figure S4 — rES cells transducted by LV-GFP. (A and B) EGFP positive colonies (line Ws-9) were picked up and injected into the blastosysts. (C and D) Cystic EBs were produced by EGFP positive Ws-9 cells. Scale Bar: 100 µm. (E and F) EGFP positive rES (line Ws-9) produced tumors after intraperitoneal injection. (1.56 MB TIF) [file pone.0002800.s007.tif]

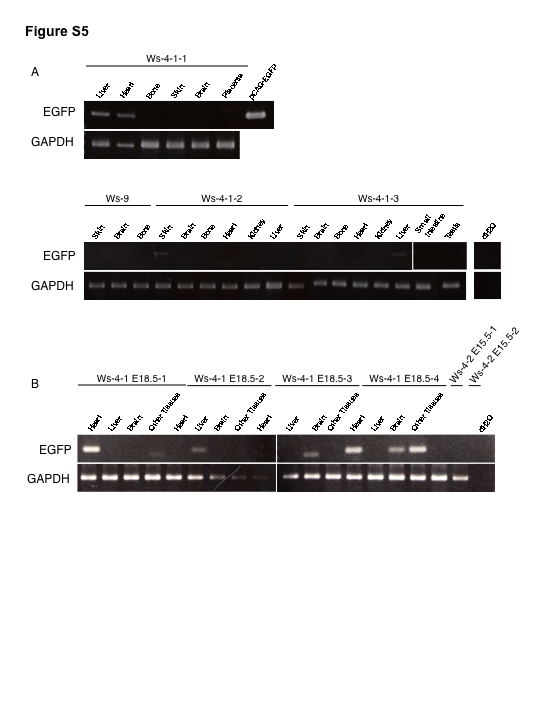

Supplement: Figure S5 — Contribution of rES cells (Ws-4-1 and Ws-9) by genotyping. (A) Three chimeric rats were obtained from Ws-4-1 and no chimeric rat was obtained from Ws-9. (B) Three chimeric embryos (E18.5) were obtained from Ws-4-1. pCAG-EGFP is a vector with EGFP gene, positive control. (1.56 MB TIF) [file pone.0002800.s008.tif]

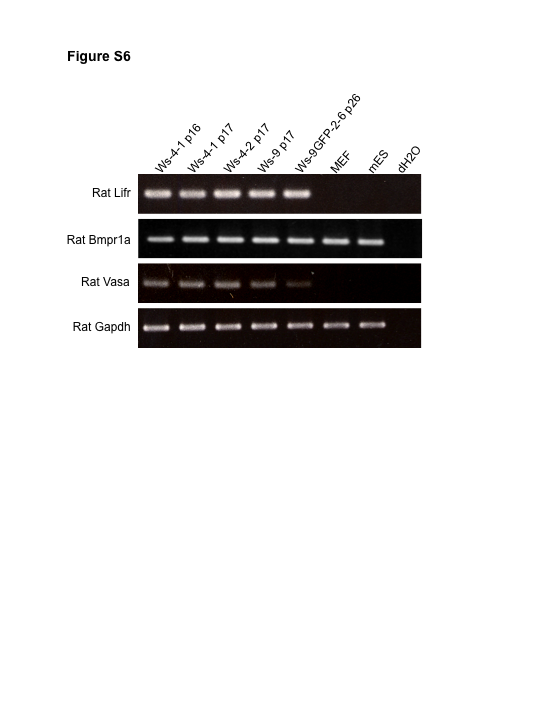

Supplement: Figure S6 — RT-PCR analysis of rat Lifr and Vasa gene expression in rES cells. Total RNA was isolated using the ISOGEN (Nippongene, Tokyo, Japan) and samples were treated with DNase I (Takara). cDNA was made from 2 µg of total RNA using Super Script III RT (Invitrogen) and oligo-dT primers (Invitrogen). cDNA was amplified with TaKaRa Ex Taq Hot Start Version (Takara, Japan) using gene specific primers. The following primers were used: rat Lifr, forward, 5′-CTGTCATTGTTGGCGTGGTA-3′ and reverse, 5′-GATTCCAGGACTTCGACGTG-3′, 30 cycles; rat Vasa, forward, 5′-TTGGGCACTCAATTCGAC-3′ and reverse, 5′-AACTTCTTCATTTCGGGTCC-3′, 32 cycles; rat Bmpr1a, forward, 5′-CCATTTCCAGCCCTACATCA-3′ and reverse, 5′-TTCCAGCGGTTAGAGACGAT-3′, 35 cycles. (1.56 MB TIF) [file pone.0002800.s009.tif]

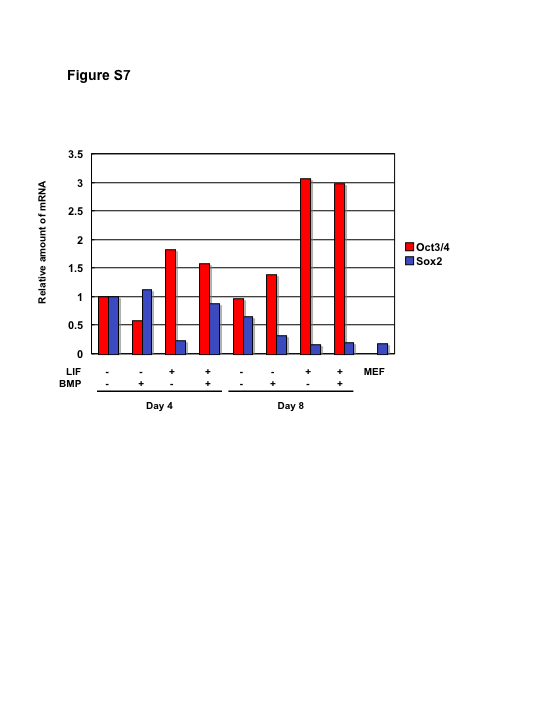

Supplement: Figure S7 — Oct3/4 and Sox2 expression analysis of rES cells in response to LIF, BMP4, or LIF plus BMP4. Ws-9 was maintained without rat LIF. The 5×10^4^/well of Ws-9 cells were plated on MEFs of gelatin coated 6 well plates in the RESM. Culture condition was divided into four groups 1) rat LIF (-) and human BMP4 (-), 2) rat LIF (-) and human BMP4 (10 ng/ml), 3) rat LIF (500 U/ml) and human BMP4 (-), 4) rat LIF (500 U/ml) and human BMP4 (10 ng/ml). After day 4, rES cells were passaged 5×10^4^/well. Real-time PCR analysis of Ws-9 p14 (day 4), p15 (day 8) and MEFs. cDNAs were used for PCR using Platinum SYBR Green qPCR SuperMix UDG (Invitrogen). Optimization of the qRT-PCR reaction was performed according to the manufacture's instructions (PE Applied Biosystems, Tokyo, Japan). The following primers were used: Oct3/4, forward, 5′-CATCTGCCGCTTCGAG-3′ and reverse, 5′-CTCAATGCTAGTCCGCTTTC-3′; Sox2, forward, 5′-CCCACCTACAGCATGTCCTA-3′ and reverse, 5′-TGGAGTGGGAGGAAGAGGTA-3′; rat Gapdh, forward, 5′-TTCAACGGCACAGTCAAGG-3′ and reverse, 5′-CATGGACTGTGGTCATGAG-3′. Transcript levels were normalized to rat GAPDH expression, and expression levels of rES cell cultured without rat LIF and human BMP4 at day 4 set to 1.0. The results present the mean of two real-time PCR analysis. Oct3/4 (Red) expression was increased in rat LIF and rat LIF + human BMP4, however, Sox2 (Blue) expression was not increased. (1.56 MB TIF) [file pone.0002800.s010.tif]

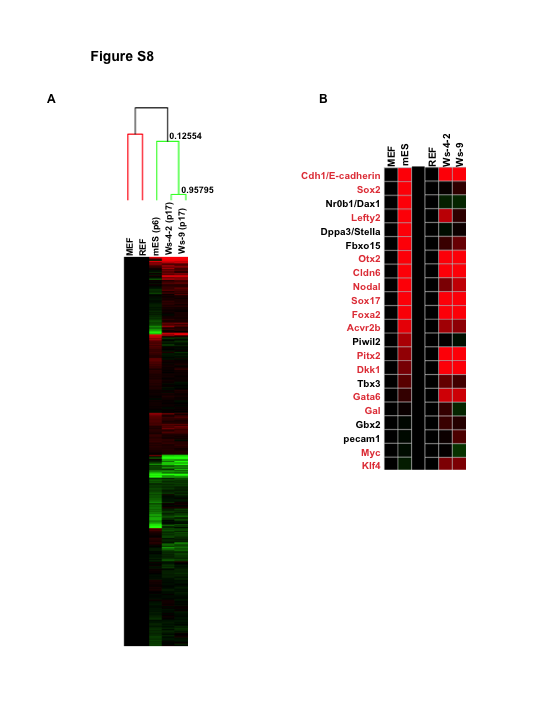

Supplement: Figure S8 — Microarray analysis and hierarchical clustering analysis. A one-color microarray-based gene expression analysis system (Agilent Technologies, Santa Clara, CA) containing 41,000 genes was used, following the manufacturer's instructions. Total RNA was extracted from MMC treated MEF, mES cells (derived from 129sv, p6), MMC treated rat embryonic fibroblast (REF, 3Y1-B,p5) [19], Ws-4-2 (p17), Ws-4-2 (p14) and Ws-9 (p17). The process of hybridization and washing was performed using a Gene Expression Wash Pack (Agilent Technologies) and acetonitrile (Sigma, Tokyo, Japan). A DNA microarray scanner (Agilent Technologies) was used for array scanning. Data normalization and cluster analysis were performed using GeneSpring GX software (Agilent Technologies). The expression level of each gene in the MMC treated MEF (for mES cells) or MMC treated REF (for rES cells) was used as a reference. Microarray data of mouse and rat was integrated via GeneSymbol, and omitted missing values. This resulted in a data matrix of 3943 genes. A hierarchical cluster was produced using an Euclidean distance calculation based on the Ward method calculation. (A) Cluster analysis was performed by sorting 3943 altered genes. Red indicates increased expression compared to levels of MMC treated MEF or MMC treated REF, whereas green means decreased expression. Correlation coefficients are indicated. This analysis of microarray data shows that there are distinct gene clusters between rES cells and mES cells. (B) The expression of genes to maintain the pluripotent state of mES cells or EpiSCs or hES cells. Gene names shown in red were detected in hES cell cultures and EpiSC using Illumina and Agilent whole-genome microarrays [34]. This microarray analysis shows that the gene expression profile of our rES cells partially resembles mES cells. (1.56 MB TIF) [file pone.0002800.s011.tif]
